# Immersive Surgical Anatomy of the Venous Drainage and Meningeal Supply of the Posterior Fossa: Anatomical Nuances and Microsurgical Management of Dural Arteriovenous Fistulas (dAVFs)

**DOI:** 10.7759/cureus.64532

**Published:** 2024-07-14

**Authors:** Andre Payman, Jorge Rios Zermeno, Viola Bartoletti, Nicolo Norri, Adib A. Abla, Roberto Rodriguez Rubio

**Affiliations:** 1 Neurological Surgery, University of California San Francisco, San Francisco, USA; 2 Neurological Surgery, Mayo Clinic, Jacksonville, USA; 3 Neurological Surgery, University of Padua, Padua, ITA; 4 Neurological Surgery, University of Ferrara, Ferrara, ITA; 5 Neurological Surgery, University of Miami, Miami, USA

**Keywords:** surgical approaches, volumetric models, microsurgery, dural arteriovenous fistula, medical education, anatomy, posterior fossa, veins, arteries, meninges

## Abstract

Dural arteriovenous fistulas (dAVFs) are anomalous connections between arteries and veins within the dura mater, involving dural sinuses, bridging veins, or emissary veins. If untreated, these lesions can result in intracranial hemorrhage. The management of posterior fossa dAVFs is challenging due to the intricate venous anatomy near the brainstem and cranial nerves. This study leverages three-dimensional (3D) technology combined with dissections to understand the anatomy and microsurgical techniques for treating infratentorial dAVFs. Five embalmed heads and one dry skull were used to meticulously document the pertinent anatomy of the infratentorial compartment. Advanced 3D technology, including 3D sculpting and structured light scanning, was employed to construct high-resolution volumetric models (VMs). Two-dimensional (2D) images of dissections and VMs illustrate key anatomical landmarks of the posterior fossa. Infratentorial dAVFs primarily involve sinuses, which are divided into groups based on their location: basal, medullary, and petrosal. Most of the arterial supply originates from the external carotid artery, especially the ascending pharyngeal artery. This is followed by meningeal branches from the internal carotid artery (ICA) and vertebrobasilar system. The surgical approaches to treat infratentorial dAVFs include the retrosigmoid and far lateral approaches and their modifications. Our study describes the relevant vascular anatomy of the infratentorial compartment, focusing on the surgical treatment of infratentorial dAVFs. In conjunction with the included interactive models, this study improves our educational capabilities regarding the intricate vascular neuroanatomical features of this region. When applied to a clinical setting, precise anatomical knowledge and VMs tools enhance surgical outcomes, reduce complications, and ultimately improve patient care.

## Introduction

Dural arteriovenous fistulas (dAVFs) are anomalous connections between arteries and veins within the dura mater, involving dural sinuses, bridging veins, or emissary veins [1-3]. The estimated incidence of cranial dAVFs is 0.16 per 100,000, and among intracranial dAVFs, posterior fossa dAVFs account for 4-12% [4]. 

If untreated, dAVFs can result in devastating intracranial hemorrhage, particularly when cortical drainage is involved, with an incidence ranging from 7% to 42% [5]. The most critical factor influencing dAVF treatment is the venous drainage pattern, specifically the presence of cortical venous drainage, indicating a more aggressive fistula [6]. 

Management of posterior fossa dAVFs is particularly challenging due to the intricate and variable venous anatomy in this region and their proximity to the brainstem and cranial nerves [7]. Furthermore, the involvement of multiple supplying systems, including the vertebrobasilar system, external carotid artery (ECA), and internal carotid artery (ICA), along with the frequent presence of leptomeningeal reflux, adds to the complexity of these cases [7]. In cases where endovascular treatment is not feasible, alternative management options, such as surgery, radiosurgery, or a combined approach, are considered [7]. The utilization frequencies of various treatment modalities for dAVFs are heterogeneous across institutions and are scarcely documented. Multiple variables, such as anatomical complexity, staff experience, and resource availability, heavily influence the therapeutic choice-making for these rare cerebrovascular lesions [7]. 

To determine the most effective treatment for dAVFs, a comprehensive understanding of the complex anatomy of each type is essential. Key considerations include the location, number, and diameter of feeding arteries, the pattern of draining veins, and the direction of blood flow. While current descriptions of microsurgical approaches often focus on classical anatomical narratives or endovascular-based images, there is a lack of emphasis on the surgical vascular anatomy of these lesions. 

This study aims to provide the anatomical basis required to treat dAVFs in the infratentorial compartment. 

This work was presented as a podium presentation at the 2024 North American Skull Base Society meeting in Atlanta, Georgia, on February 18, 2024.

## Technical report

Materials and methods

Five human cadaveric heads, embalmed and latex-injected, were prepared for anatomical dissections in a simulated surgical setting at the Skull Base and Cerebrovascular Laboratory of the University of California San Francisco (UCSF). The dissections were performed and documented, and the images were postprocessed using our previously described methodology [8]. 

A dry adult human skull and two embalmed latex-injected specimens showing relevant anatomy were reconstructed using our previously described three-dimensional (3D) structured-light scanning workflow for further analysis and representation [9]. Textured digital sculptures of vascular anatomy were created by the senior author using two-dimensional (2D) and 3D visual references (i.e., angiographies, dissections, and medical illustrations) with 3D Sculpting software for iPad OS (Nomad Sculpt 1.90, Hexanomad, Paris, France). Physically-based rendered images of vascular and osseous models were obtained with 3D software (Blender 4.0, Blender Foundation, Amsterdam, Netherlands) using a virtual studio lighting setup. 

No institutional review board (IRB)/ethics committee was required for this study. 

Virtual platform

To facilitate accessibility and interactivity, the anatomical volumetric models were uploaded to a web-based 3D model viewer (Sketchfab, Sketchfab Inc., New York, USA). Virtual scenes were designed to allow for real-time rendering, incorporating adjustments to position, lighting, materials, and filters. Strategic anatomical structures were labeled and annotated for the interactive experience. 

Vascular anatomy

The following section will discuss the main vascular anatomy relevant to infratentorial dAVFs, focusing on the transverse-sigmoid sinus, inferior petrosal sinus, and marginal sinus fistulas. Initially, we will describe the venous sinus anatomy, followed by an examination of the main arterial supply of the dura mater in the infratentorial compartment. 

Venous Anatomy

**Figure 1:**
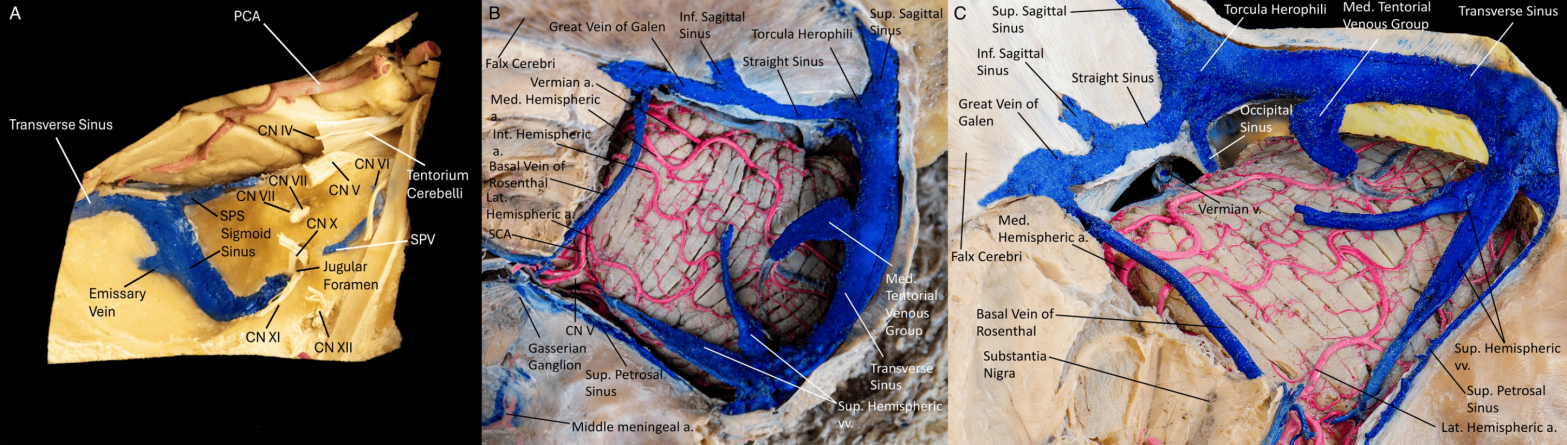
(A) Sagittal view of the neurovascular components of the posterior fossa. We can appreciate the posterior meningeal arteries and meningeal branches of the ascending pharyngeal arteries. The cranial nerves and their relationship to the transverse, sigmoid, and superior petrosal sinuses can be seen. Superior (B) and anterosuperior (C) views of the cerebellum and tentorial venous sinuses. We can appreciate the venous vasculature of the tentorial veins, great vein of Galen, superior petrosal sinus, occipital sinus, and sagittal sinuses draining into the torcula. Branches of the superior cerebellar artery are seen coursing over the superior surface of the cerebellum. The transverse sinus runs in a lateral and anterior trajectory, receiving drainage from the superior sagittal sinus, suboccipital sinus, and straight sinus. The transverse sinus and sigmoid sinus junction is marked by the opening of the superior petrosal sinus. The superior sagittal sinus and emissary veins, which pass through the mastoid foramen and condylar canal, reach the sigmoid sinus before its transition into the jugular bulb. The sigmoid sinus begins as the continuation of the transverse sinus, coursing downward in an S-shaped curve in the sigmoid sulcus on the inner surface of the temporal bone. (Published with permission of the University of California San Francisco’s Skull Base and Cerebrovascular Laboratory). a., artery; v., vein; vv., veins; Sup, superior; Inf, inferior; Med, medial; CN, cranial nerve; SPS, superior petrosal sinus; IPS, inferior petrosal sinus; PCA, posterior cerebral artery; SPV, superior petrosal vein.

This section comprehensively describes the course, draining veins, and regional anatomy of the venous sinuses within the infratentorial compartment. This includes the transverse-sigmoid sinuses, the marginal sinus, the inferior petrosal sinus, and the occipital sinus (Figure 1A). The venous anatomy pertinent to this discussion encompasses two principal categories: the sinodural veins, also known as the dural venous sinuses, and the bridging veins [6]. The sinodural veins are anatomically classified for the purpose of improving the identification of the structures related to dAVFs. Within the infratentorial fossa, the sinodural veins are delineated based on their spatial relation to the cerebellum, dividing into three distinct groups: basal, medullary, and petrosal. The basal group encompasses the transverse and sigmoid sinuses. The medullary group comprises the marginal and occipital venous sinuses, and the petrosal group includes the superior and inferior petrosal venous sinuses (Interactive Model 1).

**Video 1:** Volumetric model showing the main venous structures involved in the drainage of the posterior fossa. (Published with permission of the University of California San Francisco’s Skull Base and Cerebrovascular Laboratory).

Basal Group: Transverse-Sigmoid Sinuses

Originating at the torcular Herophili, situated at the level of the internal occipital protuberance, the transverse sinus (TS) follows the tentorium cerebelli along its external or attached margin [6]. In 60% of individuals, the right TS represents a continuation of the superior sagittal sinus (SSS) (dominant TS), draining blood from the superficial structures of the brain. Typically, the right TS is more prominent than the left in 61-78% of individuals. The left TS usually serves as a continuation of the straight sinus. Along its path, the TS receives venous drainage from the lateral temporal and basal surfaces of the temporal and occipital lobes, ultimately reaching the posterolateral portion of the petrous temporal bone to continue as the sigmoid sinus (SS) [10]. The TS consistently receives venous cranial afferents, while the SS never receives cerebral veins (Figures 1B-1C). 

The SS traverses the jugular process of the occipital bone and then bends forward to end at the jugular bulb, where it meets the jugular vein. At this point between the SS and jugular vein, a thin plate of bone, the tegmen tympani separates the upper part of the SS from the inner ear anteriorly and the mastoid antrum and air cells posteriorly [6]. 

Medullary Group: Marginal Sinus

The marginal sinus is an inconstant intradural sinus that encircles the internal rim of the foramen magnum [11]. It serves as an alternative pathway for venous drainage when the internal jugular vein is affected and may serve as the primary path in an upright position. The sinus communicates with the basilar venous plexus, occipital sinus, inferior petrosal sinus, and a network of venous channels, including the anterior condylar confluence and the condylar veins (anterior (also known as the venous plexus of the hypoglossal canal), posterior, and lateral). At its distal end, the marginal sinus drains into the vertebral (suboccipital) venous plexus and anterior condylar confluence [11,12]. 

The maximum vertical height of the marginal sinus is located where the spinal accessory nerve (CN XI) crosses it, tapering to its lowest height in the anterior and posterior portions of the foramen magnum. Like the ICA in the cavernous sinus, the vertebral artery can traverse through the marginal sinus [12]. 

Medullary Group: Occipital Sinus

**Figure 2:**
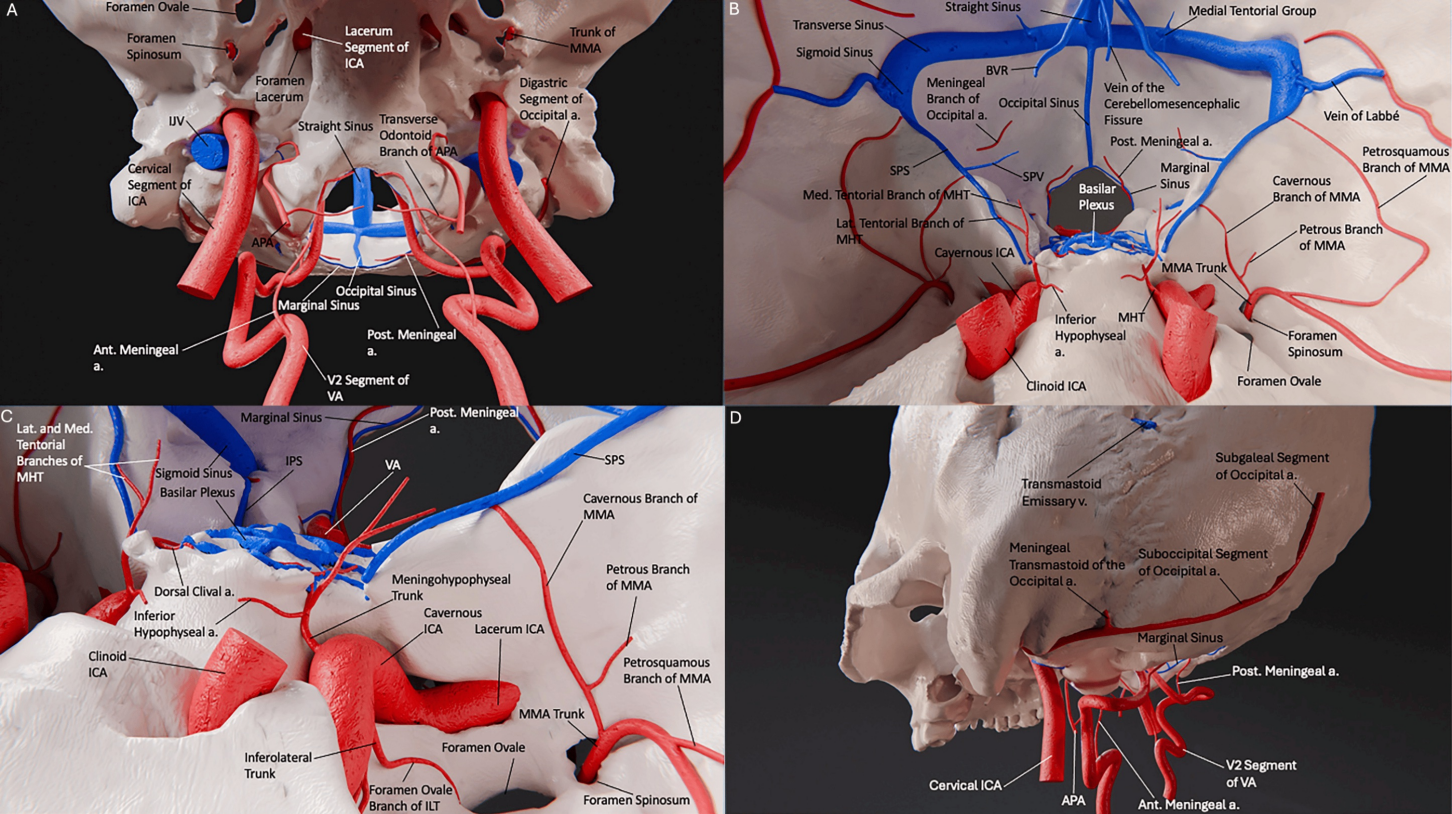
3D model render of the arteriovenous anatomy of the posterior skull base. (A) Anteroinferior view of the exocranial skull base. We can appreciate the course of the extracranial internal carotid and vertebral arteries and their branches, and the course of the occipital sinus as it travels inferiorly. This sinus communicates superiorly with the confluence of sinuses and drains to the marginal sinus or the vertebral venous plexus. (B) View of the middle and posterior fossae of the skull base with relevant vascular structures. We can appreciate the course of the ICA as it exits the petrous bone and its branches, and the course of the SPS on the petrous bone. The SPS originates anteriorly in the cavernous sinus and follows a posterior and lateral trajectory towards the transverse-sigmoid junction near the base of the petrous bone. The principal tributary of the SPS is the petrosal vein, also referred to as the superior petrosal complex. This vein is a critical drainage pathway for the inferior cerebral veins, the anterior portion of the cerebellum, the brainstem, and the nearby cranial nerves. (C) Posterolateral view of the exocranial skull. We can appreciate the extracranial arteries, with the course and transosseous branch of the occipital artery shown. We can appreciate the inferior petrosal sinus as it courses within the inferior petrosal sulcus at the junction of the petrous bone with the basilar portion of the occipital bone and terminates at the anteromedial portion of the jugular foramen. It drains the cavernous sinus, receiving venous drainage from the medulla oblongata, pons, and inferior cerebellar surface along its route. (D) Anterosuperior view of the medial and posterior fossae of the skull base with relevant vascular structures. We can appreciate the infratentorial venous anatomy and its relationship with the osseous and arterial structures in the region. The anterior meningeal artery arises from the vertebral artery at the level of C2, courses through the intervertebral foramen, and continues superiorly toward the midline. (Published with permission of the University of California San Francisco’s Skull Base and Cerebrovascular Laboratory). Post., Posterior; Ant., Anterior; Sup., Superior; Inf., Inferior; Lat., lateral; Med., medial; a., artery; ICA, internal carotid artery; APA, ascending pharyngeal artery; VA, vertebral artery; MMA, middle meningeal artery; ILT, inferolateral trunk; MHT, meningohypophyseal trunk; IJV, internal jugular vein; BVR, basal vein of Rosenthal; SPS, superior petrosal sinus; SPV, superior petrosal vein; C2, cervical vertebra 2.

The occipital sinus is a small venous sinus located in the posterior midline of the posterior cranial fossa. It forms when the venous channels from the occipital plexus - most prominent in the second trimester of gestation - regress to form this midline structure. Occipital sinus presence varies, with reports indicating its existence in 64.5% to 81.7% of adults and exhibiting multiple anatomical variations, such as duplication and oblique occipital sinus (Figure 2A) [13,14].

Petrosal Group: Superior Petrosal Sinus

The superior petrosal sinus (SPS) is a venous channel situated along the attached margin of the tentorium within the superior petrosal sulcus. Notably, the SPS is more pronounced in its posterolateral part and diminishes in size as it nears the petrous apex (Figure 2B) [10]. 

Petrosal Group: Inferior Petrosal Sinus

The inferior petrosal sinus (IPS) is a venous structure originating from the posteroinferior aspect of the cavernous sinus. Numerous anastomotic channels exist between the IPS and the basilar venous plexus, vertebral venous plexus, pterygoid venous plexus, and epidural veins [15]. 

Multiple venous communicating channels commonly exist between the left and right IPS at the posterosuperior cavernous sinus. This communication extends along the clivus for a variable distance but constantly extends at least to the level of Dorello's canal. The sheet of collateral venous communication gradually thins until a highly variable number of small, collateral venous communicators travel over the clivus to interconnect the left and right IPS - these are part of the basilar plexus. Dorello's canal traverses the IPS at a slight medial-to-lateral angle [13]. 

Bridging Veins and Leptomeningeal Venous Drainage

Bridging veins (BVs) connect the pial venous system with the dural sinuses and are commonly involved in the dAVFs, particularly if direct cortical venous drainage is present [16]. Shunts involving bridging veins affect the dural segment, consistently showing direct leptomeningeal drainage (retrograde flow). This could result from partial or complete thrombosis of the affected sinus, leading to symptoms when the connection between the sinus and the BV becomes occluded [16]. 

The BVs draining the posterior fossa collect into three groups: superior or galenic, which drains the tentorial surface, cerebellomesencephalic fissure, and superior half of the roof of the fourth ventricle into the vein of Galen; anterior or petrosal, which drains the petrosal surface of the cerebellum, cerebellopontine and cerebellomedullary fissures, the inferior half of the roof of the fourth ventricle, and the anterior and lateral pons and medulla into the petrosal sinuses; and posterior or tentorial, which drains the suboccipital surface into the torcula. These BVs include the superior vermian, superior hemispheric, tentorial bridging, superior petrosal, and inferior petrosal veins [16]. 

Arterial anatomy

In the next section, we will describe the arteries that provide vascular supply to the posterior fossa dura mater. The arterial supply can be categorized into two main groups according to its origin: extracranial, originating from the external carotid artery, and intracranial, deriving from the internal carotid artery and vertebrobasilar system (Interactive Model 2).

**Video 2:** Volumetric model showing the main arteries involved in the meningeal blood supply to the posterior fossa. (Published with permission of the University of California San Francisco’s Skull Base and Cerebrovascular Laboratory). APA, ascending pharyngeal artery

Arteries Originating From External Carotid Artery 

**Figure 3:**
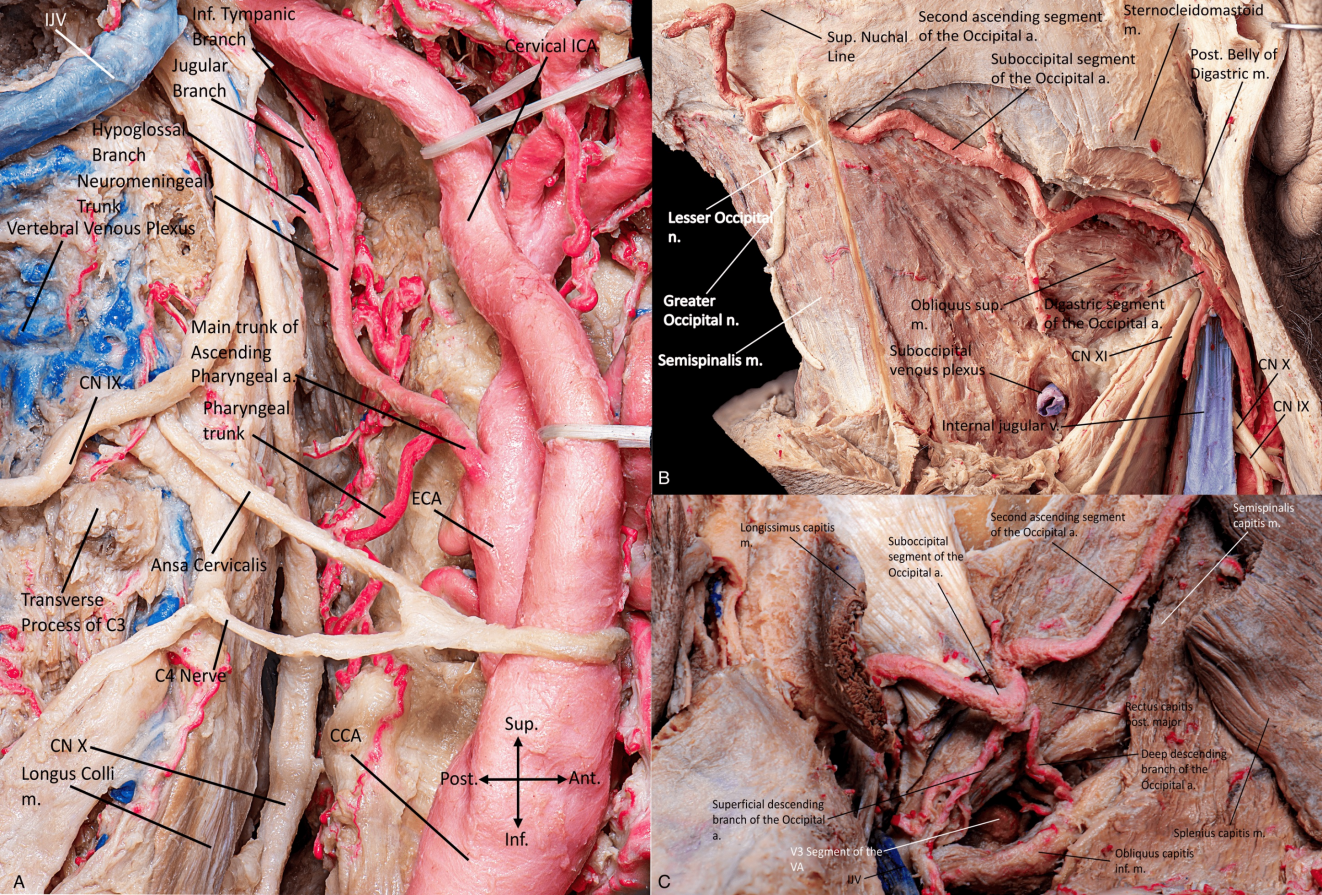
(A) Lateral view of the right lateral pharyngeal space, where we can appreciate the common carotid artery bifurcation, the course and branches of the ascending pharyngeal artery, and lower cranial nerves. The ascending pharyngeal artery arises from the posterior or medial surface of the proximal external carotid artery, near the common carotid bifurcation, and adjacent to the origins of the occipital and lingual arteries. It may also emerge from the occipital artery, lingual artery, cervical arteries, or as a common trunk with the occipital artery named the auriculo-occipital trunk. The ascending pharyngeal artery (APA) follows an ascending vertical course, slightly convex anteriorly, between the internal and external carotid arteries along the posterolateral wall of the pharynx. Posterior view of the right superficial (B) myofascial neck anatomy illustrating the course of the occipital artery and related neural and muscular structures. The lower cranial nerves are visible in relation to the course of the occipital artery and internal jugular vein. The right deep (C) myofascial anatomy allows for the visualization of the occipital triangle as well as the descending branches of the occipital artery forming an anastomosis with the vertebral artery. The occipital artery usually originates from the posterior surface of the ECA at the level of the mandible, proximal to the origin of the facial artery. It may also arise as a single trunk with the ascending pharyngeal artery or from the internal carotid artery or vertebrobasilar system. It has cutaneous, muscular, and neuromeningeal branches, and its territory may be extensive: it usually supplies the muscles of the upper cervical region, meninges of the posterior fossa, peripheral nerves (C1 and C2), and the scalp. (Published with permission of the University of California San Francisco’s Skull Base & Cerebrovascular Laboratory). n., nerve; a., artery; m., muscle; v., vein; Sup., superior; Post., posterior; Inf., inferior; Ant., anterior; C, cervical; CN, cranial nerve; V, vertebral; VA, vertebral artery; CCA, common carotid artery; ECA, external carotid artery; ICA, internal carotid artery; IJV, internal jugular vein; C1, cervical vertebra 1; C2, cervical vertebra 2.

Ascending pharyngeal artery (APA): The APA courses superiorly prior to bifurcating into the pharyngeal and meningeal trunks. The pharyngeal trunk runs adjacent to the pharynx, ending at the base of the petrous bone near the carotid canal. The meningeal trunk courses posteriorly to the base of the skull near the occipital condyle [17]. The branches of the ascending pharyngeal system include the pharyngeal branches, prevertebral branch, musculospinal branch, inferior tympanic branch, and neuromeningeal trunk (Figure 3A) [17,18]. 

The pharyngeal branches are traditionally described as inferior, middle, and superior (Eustachian branch) and often originate from a single trunk. They supply the medial and paramedial mucosa of the naso- and oropharynx, anastomosing in the midline with contralateral branches [14]. A significant intra- to extracranial anastomosis with the ICA comes from a branch arising from the superior pharyngeal branch. This branch ascends and enters the carotid canal, continuing across the foramen lacerum, and accompanies the ICA, finally anastomosing with the inferolateral trunk [14]. 

The inferior tympanic branch may arise from the anterior (pharyngeal) or posterior (neuromeningeal) division of the APA or as a third terminal branch (trifurcation). It accompanies the tympanic branch of the ninth cranial nerve within the tympanic cavity [14]. 

The neuromeningeal branches originate from the apex of the posterior branch and continue​​​​​​ posteriorly toward the anterior condylar canal. When viewed from a lateral projection, these branches follow a bayonet course: concave inferiorly extracranially and anterosuperiorly intracranially [18]. The neuromeningeal trunk gives rise to the hypoglossal branch, the jugular branch, and a prevertebral branch [14]. 

The hypoglossal branch, entering through the hypoglossal canal, supplies the hypoglossal nerve, dura of the posterior cranial fossa, foramen magnum, and clivus [17]. The ascending branch to the clivus may anastomose​​ at the dorsum sella with the medial clival artery from the ipsilateral dorsal meningeal artery, a branch of the meningohypophyseal trunk of the ICA [17]. The hypoglossal branch also gives a descending artery that anastomoses with the vertebral artery's anterior meningeal artery branch, forming the arterial arcade of the odontoid process [17]. 

The jugular branch enters through the jugular foramen along with lower cranial nerves: glossopharyngeal (CN IX), vagus (CN X), and CN XI, dividing into medial and lateral branches. The lateral branch courses along the sigmoid sinus, anastomosing with the jugular branch of the occipital artery, and supplies the dura mater of the cerebellopontine angle [14]. The medial branch follows the course of the IPS, supplying abducens nerve (CNVI), and anastomoses with the medial branch of the lateral clival artery, subarcuate artery, petrosquamosal branch of the middle meningeal artery, and the mastoid branches of the occipital artery [17]. 

The meningeal territory of the APA is highly variable, ranging from the foramen magnum and jugular foramen to extending posteriorly to the meninges of the occipital region and anteriorly to the meninges of the petrous bone and clivus [18]. 

Occipital artery: The occipital artery consists of three segments: ascending (digastric), horizontal (suboccipital), and second ascending (occipital or subgaleal) (Figures 3B-3C) [18]. 

The ascending segment arises from the ECA and travels obliquely backward and ​​medial to the posterior belly of the digastric muscle in the retro-styloid space. It crosses the anterior surface of the ICA and runs laterally to it, as well as the internal jugular vein, vagus nerve, accessory nerve, and hypoglossal nerve. The ascending segment includes muscular branches to the sternocleidomastoid, digastric, rectus lateralis, and obliques, as well as the stylomastoid artery. 

The horizontal segment is related to the mastoid process, crossing the posterior border of the external auditory canal, and running in a sulcus on the temporal bone called the occipital groove. It is bordered laterally by the insertion of the posterior belly of the digastric muscle and medially by the superior oblique muscle. The horizontal segment gives rise to an inconstant auricular branch to the mastoid region, a descending muscular branch, and a meningeal branch that enters the skull through the mastoid foramen. 

The third segment (second ascending) marks the termination of the occipital artery, where it becomes vertical. It begins by piercing the fascia between the trapezius and the sternocleidomastoid muscles after passing through the insertion of the splenium capitis below the superior nuchal line, after passing either superficially or deep to the longissimus capitis. This segment provides multiple scalp branches and muscular branches [18]. 

Two branches of the occipital artery, originating from the second and third segments, supply the dura mater of the posterior fossa: the artery of the falx cerebelli and the mastoid branch. 

The mastoid branch (also called the transmastoid or artery of the mastoid foramen) arises from the horizontal segment. It enters the cranial cavity through the transmastoid venous emissary foramen (mastoid foramen), emerging at the level of the superior edge of the sigmoid sinus. The mastoid branch can be divided into three groups: a descending branch to the jugular foramen, an ascending (superior) branch to the posterosuperior aspect of the cerebellopontine angle, and a posteromedial branch to the dura of the cerebellar fossa. The mastoid branch has anastomoses with the petrosquamous branch of the middle meningeal artery (MMA), the APA, and the vertebral artery (Interactive Model 3) [14]. 

**Video 3:** Volumetric models showing the main vascular structures relevant to infratentorial dAVFs and their relationship with cranial anatomy. (Published with permission of the University of California San Francisco’s Skull Base and Cerebrovascular Laboratory). dAVFs, dural arteriovenous fistulas

**Figure 4:**
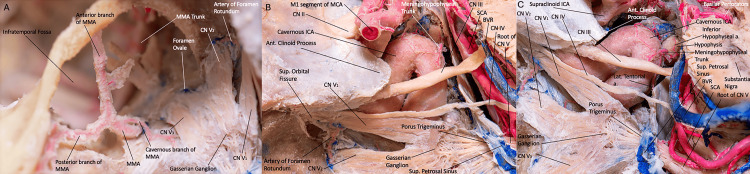
(A) Superior view of the left infratemporal fossa, illustrating the neurovascular structures of this region. We can appreciate the origin and branches of the MMA in relation to the trigeminal nerve. The MMA is the second ascending branch of the internal maxillary artery, arising from its first segment (mandibular) behind the condylar process of the mandible. (B, C) Overview of the cavernous ICA illustrating the neurovascular structures of the region. We can appreciate the course of the superior petrosal sinus anterior to the midbrain, and the origin of the meningohypophyseal trunk from the ICA. The meningohypophyseal trunk is the most consistent and largest branch of the cavernous ICA. (Published with permission of the University of California San Francisco’s Skull Base and Cerebrovascular Laboratory). ant., anterior; Lat., lateral; Sup., superior; a., artery; ICA, internal carotid artery; CN, cranial nerve; SCA, superior cerebellar artery; BVR, basal vein of Rosenthal; a., artery; CN, cranial nerve; MMA, middle meningeal artery.

MMA branches - petrous and petrosquamous: The MMA enters the cranial cavity through the foramen spinosum and then courses laterally towards the greater sphenoid wing, where it typically divides into anterior and posterior branches (Figure 4A). 

Before its division, the trunk of the MMA gives rise to three principal posterior branches: petrosquamous, middle posterior, and posterosuperior branches. The petrosquamous (posteroinferior, squamotemporal) branch arises near the junction of the cranial base and the convexity, supplying the tentorium, transverse sinus, torcula, and superior petrosal sinus. Occasionally, it may extend to supply the dura of the posterior fossa, including the transverse and sigmoid sinuses. It may anastomose in the upper clivus and posterior petrous surface with the dorsal meningeal and subarcuate arteries. Distally, at the level of the junction of the sigmoid, transverse, and superior petrosal sinuses, it may anastomose with meningeal branches of the occipital, vertebral, and ascending pharyngeal arteries (Figure 2D) [17,18]. 

The posterior auricular artery can have transosseous branches that cross through the mastoid, and this may supply CN VII, as well as cortical, bony, and meningeal structures [14]. 

Arteries Originating From Internal Carotid Artery 

Meningohypophyseal trunk: The meningohypophyseal trunk arises from the medial face of the carotid, lateral to the dorsum sellae, and gives rise to the tentorial (medial and lateral), inferior hypophyseal, and dorsal meningeal arteries. 

The lateral tentorial artery passes posteriorly, superiorly, and slightly laterally to enter the tentorium along its attachment. There, it anastomoses with the petrosal and petrosquamous branches of the middle meningeal artery and with the lateral branch of the dorsal meningeal artery. 

The dorsal meningeal artery (also called the lateral clival artery) passes through the cavernous sinus in a posterior trajectory to supply the dura of the dorsum sellae and clivus. It divides into medial and lateral branches. The medial branch runs below the petrosphenoid ligament, anastomosing with the clival ramus of the jugular branch of the APA. The lateral branch courses above Meckel's cave, supplying the superior petrosal sinus, petrous ridge dura, and the arterial arcade of the tentorium cerebelli. It anastomoses with branches of the MMA (Figures 4B-4C). 

Vertebrobasilar System 

**Figure 5:**
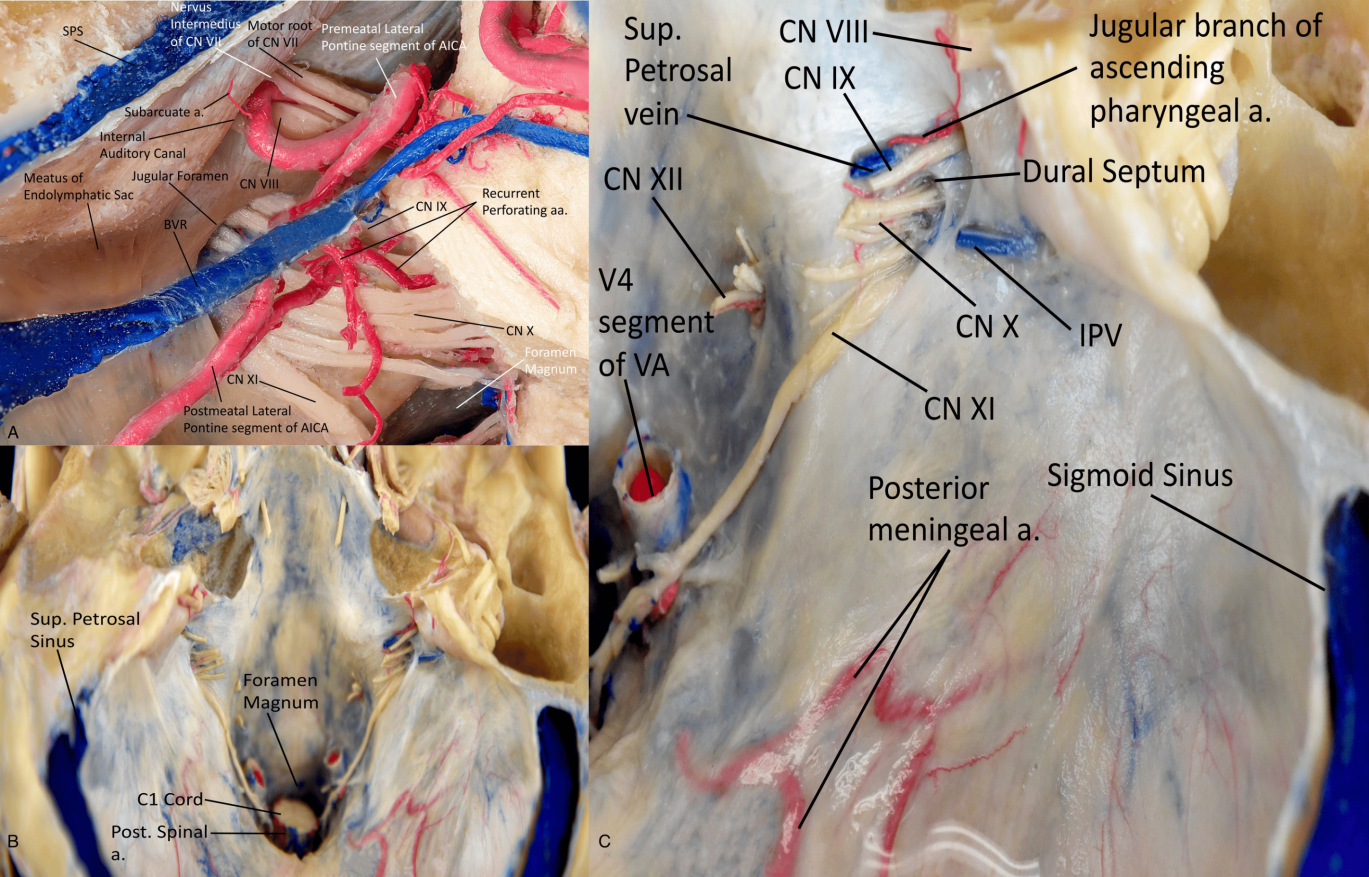
(A) View of the neurovascular structures entering the left skull base. We can appreciate the course of the subarcuate artery as it enters the subarcuate fossa and canal. The subarcuate artery arises from the cerebellolabyrinthine trunk of the AICA. It typically originates from the lateral pontine segment and pierces the dura covering the subarcuate fossa, entering the subarcuate canal. The course of the lateral pontine segment of the AICA can be seen, in addition to the seventh and eighth cranial nerves entering the internal auditory meatus. The lower cranial nerves can be seen entering the jugular foramen. Superior (B) and close-up right-side (C) perspectives of the posterior cranial fossa and its neurovascular components. We can appreciate the course of the posterior meningeal artery, as well as the various cranial nerves entering the jugular and hypoglossal foramina. The posterior meningeal artery arises from the vertebral arteries in its atlantic segment (V3) near the dural entrance, ascending in a posterosuperior direction parallel to the internal occipital crest. (Published with permission of the University of California San Francisco’s Skull Base and Cerebrovascular Laboratory). Post., posterior; Sup., superior; Inf., inferior; a., artery; C, cervical; V, vertebral; CN, cranial nerve; VA, vertebral artery; AICA, anterior inferior cerebellar artery.

Subarcuate artery: The subarcuate artery participates in dural irrigation at the cerebellopontine angle, the superolateral edge of the internal acoustic meatus, and the posterior surface of the petrous bone. It anastomoses with branches of the MMA and with mastoid branches of the occipital artery (Figure 5A). 

Anterior and posterior meningeal arteries: The anterior meningeal artery joins its contralateral paired artery to create a vascular arch at the level of the apex of the dens. It anastomoses intracranially with the hypoglossal branch of the ascending pharyngeal artery (Figure 2D). 

The posterior meningeal artery bifurcates at the level of the external occipital protuberance, anastomosing with meningeal branches of the occipital and middle meningeal arteries. It may also originate from the occipital artery, hypoglossal branch of the APA, ICA, and posterior inferior cerebellar artery (PICA) (Figures 5B-5C). 

Surgical approaches

In the treatment of infratentorial dAVFs, various surgical approaches are employed. The two main approaches used are the retrosigmoid and its variant, the extended retrosigmoid approach, as well as the far lateral approach. In this anatomical study, we illustrate the continued utility of these approaches to treating various infratentorial dAVFs (Interactive Model 4) [19].

**Video 4:** Volumetric models showing the main surgical exposure utilized for the microsurgical treatment of infratentorial dAVFs and their relevant anatomy. (Published with permission of the University of California San Francisco’s Skull Base and Cerebrovascular Laboratory). dAVFs, dural arteriovenous fistulas

*Retrosigmoid and Extended Retrosigmoid Approach* 

**Figure 6:**
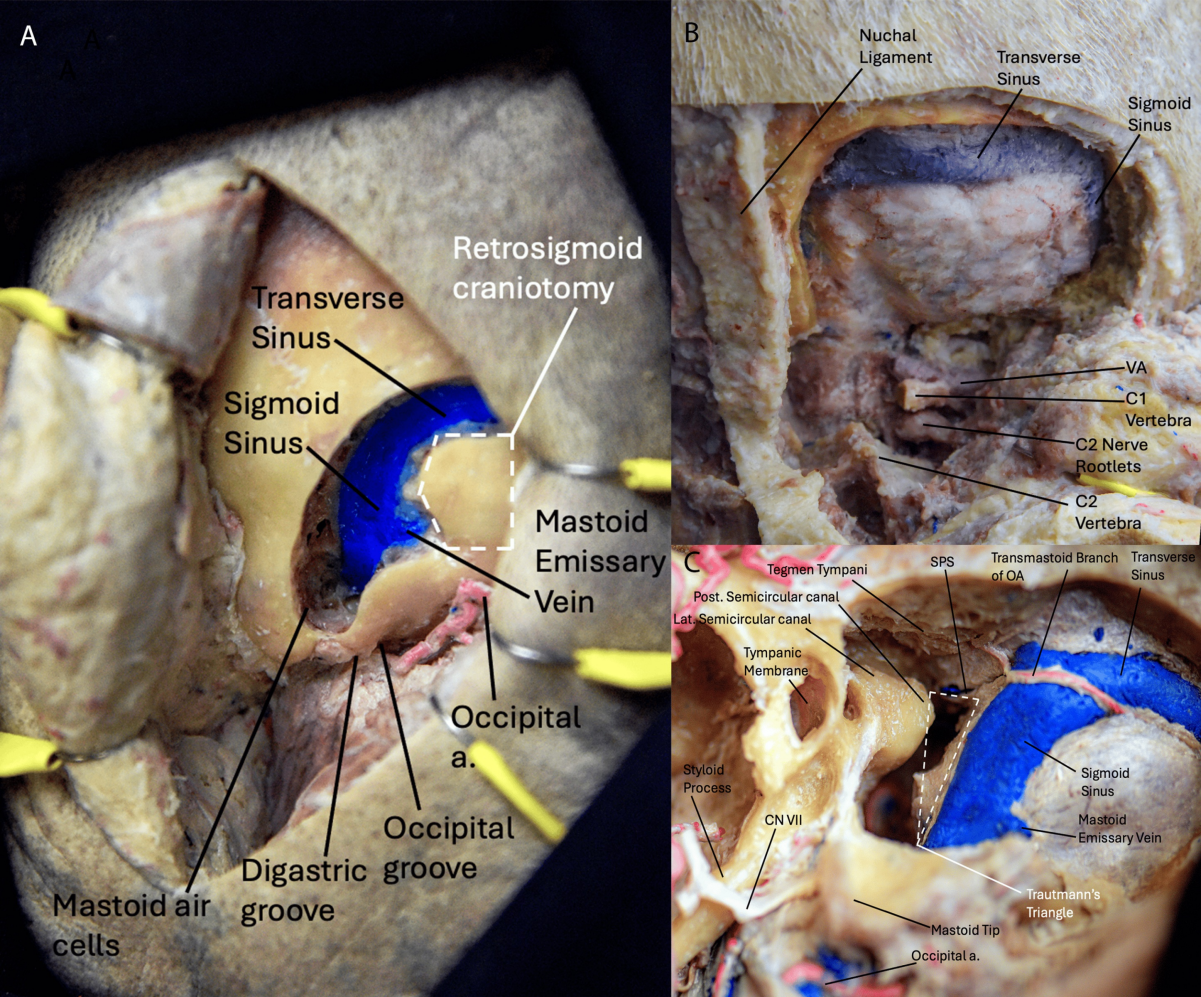
Surgical approaches to the posterior fossa. (A) Posterolateral view of the retrosigmoid approach. It provides a wide corridor to the cerebellopontine angle, internal auditory meatus, and petroclival area with minimal soft tissue dissection. We can appreciate the venous structures, including the mastoid emissary vein. (B) Posterior view highlighting the structures visible in the far lateral approach. We can appreciate the structures accessed by this approach, including the transverse and sigmoid venous sinuses. (C) View of the presigmoid or retrolabyrinthine approach. We can appreciate the structures accessed by this approach, including the sigmoid sinus and transmastoid branch of the occipital artery. (Published with permission of the University of California San Francisco’s Skull Base and Cerebrovascular Laboratory). Post., posterior; Lat., lateral; a., artery; CN, cranial nerve; OA, occipital artery, SPS, superior petrosal sinus; VA, vertebral artery; C1, cervical vertebra 1.

The retrosigmoid approach is the most frequently utilized approach to the posterior fossa. In the extended retrosigmoid approach, bone removal is extended to expose the TS and the SS (Figure 6A) [19].

When the TS is involved, a supratentorial extension of the craniotomy must be performed. For cases involving the SS, a posterior petrosectomy might be necessary to expose the anterior edge of the sinus.

Far Lateral Approach 

The far lateral approach is a classic skull base technique that provides access to the craniocervical junction and lower clivus, inferior cranial nerves (CN IX to XII), distal vertebral artery, inferior basilar trunk, and PICA (Figure 6B) [20]. 

*Presigmoid/Retrolabyrinthine Approach* 

A deeper extradural exposure to the regional vascular anatomy has also been described for the treatment of infratentorial DAVFs via the presigmoid retrolabyrinthine approach (Figure 6C) [19].

## Discussion

This study provides a comprehensive review of the relevant arterial and venous anatomy of the infratentorial compartment, in addition to the surgical approaches to the region, specifically within the context of dAVFs using 3D models to visualize the complex anatomy interactively.

dAVFs are typically managed using endovascular therapy through arterial embolization or transvenous occlusion. However, surgical intervention, radiosurgery, or a combination may be employed when endovascular treatment is not feasible. The choice of treatment strategy depends on the localization of the shunt and venous angioarchitecture of the dAVF. These factors are described and categorized by the Djindjian, Borden, Cognard, and D’Aliberti classification systems for dAVFs. Specifically, the presence of a bridging vein shunt with reflux and stasis (high-risk fistulas) is associated with the most aggressive clinical presentation [2,18,19]. 

Surgical treatment of dAVFs is considered a safe and effective approach, either as a standalone method or in combination with other treatments. Surgical intervention is usually reserved for cases where endovascular treatment has failed or when safe access to the dAVF is challenging to achieve safely [19]. Difficulties in endovascular management may arise due to factors such as the presence of numerous or tortuous arterial feeders unsuitable for embolization, arterial feeder branches extending into normal structures near or at the fistulous connection, long and tortuous feeders that are difficult to navigate, incomplete fistula obliteration with additional feeders recruited, or recanalization of previously embolized vessels. 

The fundamental principle of surgical treatment involves isolating the affected sinus or sinuses from the dura mater [19]. Historically, surgical treatment of dAVFs involved direct ligation of dural arterial feeders and arterialized draining veins, complete resection of the dAVF with all involved dura, packing, occluding, or resecting the dural sinus, as well as skeletonization and arterial devascularization of the offending dural sinus, or a combination of these techniques. However, these methods carried ​​​​high morbidity and mortality rates. As a result, a more targeted surgical approach has become the gold standard for high-grade dAVF, which involves selectively disconnecting the cortical or leptomeningeal venous drainage by ligating the arterialized vein where it emerges from the fistula. For low-grade fistulas, fistula excision with resection of the affected sinus represents the optimal surgical strategy. ​​Additionally, any feeding dural arteries encountered during the surgical exposure should be coagulated and divided [19].

dAVFs 

A variety of dAVFs are described in the following section, with the detailed vascular, skull base, and infratentorial space described and categorized in addition to the relevant surgical approach (Table 1). 

**Table 1:** Summary of the infratentorial dural arteriovenous fistulas, relevant anatomy, and microsurgical exposures. MHT, meningohypophyseal trunk; MMA, middle meningeal artery; OA, occipital artery; APA, ascending pharyngeal artery; VA, vertebral artery.

Skull Base Anatomy	Infratentorial Space	Affected Sinus	Arterial Supply	Approach
Medial segment: from the internal occipital protuberance of the occiput to the mastoid temporal bone. Lateral segment: from mastoid temporal bone along the petro-occipital suture to the carotico-jugular crest.	Posterior supracerebellar-infratentorial	Transverse-sigmoid sinus	Tentorial and dorsal meningeal arteries (MHT); Petrous or petrosquamous branches (MMA); Transosseous and transmastoid branches (OA)	Extended retrosigmoid approach (with or without sinus skeletonization)
Anterior foramen magnum: anterior to the occipital condyles. Posterior foramen magnum: posterior to the occipital condyles.	Spinomedullary junction	Marginal sinus	Neuromeningeal branch (APA); Occipital artery; Meningeal branches (VA)	Far lateral approach (with or without paracondylar extension)
Anterior segment of the petrous bone: from the petrous apex to the internal auditory meatus. Posterior segment of the petrous bone: from internal auditory meatus to jugular fossa.	Cerebellopontine	Inferior petrosal sinus; Superior petrosal sinus	Clival branches (MHT); MMA; Vertebrobasilar system; APA	Retrosigmoid approach/extended retrosigmoid/retrolabyrinthine Approach

Transverse-Sigmoid Sinus dAVFs

Transverse-sigmoid sinus fistulas may have a large number of meningeal tributaries from the external carotid (main supply), internal carotid, and vertebral arteries. The abnormal connection is commonly localized at the borderline between the junction of the transverse and sigmoid sinuses [19]. 

Transverse-sigmoid sinus dAVFs are supplied by branches of the internal carotid artery and external carotid artery. These include the tentorial and dorsal meningeal arteries that arise from the meningohypophyseal trunk of the cavernous ICA, petrous or petrosquamous branches of the MMA, and transosseous and transmastoid branches from the occipital artery. 

Marginal Sinus (Foramen Magnum/Hypoglossal Canal) dAVFs

Marginal sinus dAVFs involve shunts at the marginal sinus and condylar veins and are anatomically complex due to the variability in the venous drainage of the craniocervical junction. 

These fistulas are mainly supplied by the neuromeningeal branch of the ascending pharyngeal artery, the occipital artery, and meningeal branches from the vertebral artery; in 62.5% of cases, they can have contralateral supply [2]. 

A far lateral approach, with consideration of a paracondylar extension, is typically employed to expose the foramen magnum and the jugular bulb. 

*Superior Petrosal Sinus dAVFs* 

Superior petrosal sinus dAVFs consistently drain infratentorially into the petrosal vein. These dAVFs have almost constant cortical drainage and frequently cause venous hypertension in the brainstem, cerebellum, or upper cervical spinal cord. About half of these dAVFs are Borden Type II fistulae, with drainage into the superior petrosal vein, lateral mesencephalic vein, basal vein of Rosenthal, or cerebellar hemispheric vein. These dAVFs are often supplied by the branches of the meningohypophyseal trunk or MMA [19]. 

Inferior Petrosal Sinus dAVFs

Inferior petrosal sinus dAVFs are supplied by branches of the vertebrobasilar system and the MMA. Other branches involved are the clival branches from the meningohypophyseal trunk of the ICA and the APA. Surgical resection of inferior petrosal sinus fistulas is typically performed using a retrosigmoid approach [19]. 

Limitations 

Our study is limited by a few factors, namely a lack of clinical studies and a lack of generalizability. One significant limitation is the lack of direct clinical studies or patient data. We present anatomical dissections and 3D reconstructions, which offer detailed insights into the vascular anatomy relevant to dAVFs, but do not provide information on the clinical presentation, patient outcomes, or the practical challenges encountered during surgical treatment of these lesions. Furthermore, given that our study is based on a limited number of cadaveric specimens, there might be variations in vascular anatomy that we were unable to examine or detail. These anatomical differences can affect the generalizability of our findings to broader clinical practice.

## Conclusions

Endovascular management of dAVFs continues to evolve and has become the mainstay of treatment. However, there are cases where endovascular treatment is not feasible or may not yield optimal results. In such instances, microsurgical intervention remains a valuable alternative, highlighting the importance of surgical anatomical complexities related to these fistulas. Modern photography techniques and 3D technology provide an enhanced spatial representation of arterial and venous anatomy relevant to the management of dAVFs. These representations are crucial in visualizing the infratentorial angioarchitecture of regional dural supply and drainage and their relationship to extra- and intracranial structures.

Regardless of the therapeutic modality used for the management of posterior fossa dAVFs, invariably, the combination of the presented anatomical content can be helpful in the understanding, planning, and execution of interventions, as it provides elements to enhance topological precision and accuracy. 
